# P_RNA_scaffolder: a fast and accurate genome scaffolder using paired-end RNA-sequencing reads

**DOI:** 10.1186/s12864-018-4567-3

**Published:** 2018-03-02

**Authors:** Bai-Han Zhu, Jun Xiao, Wei Xue, Gui-Cai Xu, Ming-Yuan Sun, Jiong-Tang Li

**Affiliations:** 1 Key Laboratory of Aquatic Genomics, Ministry of Agriculture, CAFS Key Laboratory of Aquatic Genomics and Beijing Key Laboratory of Fishery Biotechnology, Chinese Academy of Fishery Sciences, Beijing, 100141 China; 2 College of Fisheries and Life Science, Shanghai Ocean University, Shanghai, 201306 China; 3 College of Marine Science, Zhejiang Ocean University, Zhoushan, 316022 China

**Keywords:** RNA-sequencing, Genome scaffolding, Single-molecule sequencing, circRNA

## Abstract

**Background:**

Obtaining complete gene structures is one major goal of genome assembly. Some gene regions are fragmented in low quality and high-quality assemblies. Therefore, new approaches are needed to recover gene regions. Genomes are widely transcribed, generating messenger and non-coding RNAs. These widespread transcripts can be used to scaffold genomes and complete transcribed regions.

**Results:**

We present P_RNA_scaffolder, a fast and accurate tool using paired-end RNA-sequencing reads to scaffold genomes. This tool aims to improve the completeness of both protein-coding and non-coding genes. After this tool was applied to scaffolding human contigs, the structures of both protein-coding genes and circular RNAs were almost completely recovered and equivalent to those in a complete genome, especially for long proteins and long circular RNAs. Tested in various species, P_RNA_scaffolder exhibited higher speed and efficiency than the existing state-of-the-art scaffolders. This tool also improved the contiguity of genome assemblies generated by current mate-pair scaffolding and third-generation single-molecule sequencing assembly.

**Conclusions:**

The P_RNA_scaffolder can improve the contiguity of genome assembly and benefit gene prediction. This tool is available at http://www.fishbrowser.org/software/P_RNA_scaffolder.

**Electronic supplementary material:**

The online version of this article (10.1186/s12864-018-4567-3) contains supplementary material, which is available to authorized users.

## Background

In a genome sequencing project, identifying genes is fundamental to functional study and comparative analysis. Although mate-pair libraries and long single-molecule reads facilitate generating high-quality assemblies, it is difficult to recover the complete structures of all genes. Therefore, novel scaffolding methods are necessary to solve this difficulty. Transcribed genomes generate different types of RNAs, such as mRNAs and long non-coding RNAs [1]. These widespread RNAs can be used to scaffold genomes and complete the structures of transcribed regions. For low-quality genome assemblies, many approaches have been generated to increase the continuity of the gene regions using proteins [2] or transcripts [3] as guides. Since RNA-sequencing technology captures both mRNAs and non-coding RNAs [4], using RNA-sequencing reads to scaffold genomes can rebuild transcribed regions. We previously developed L_RNA_scaffolder, which scaffolded genomes using long single-end RNA-sequencing reads or assembled transcripts from paired-end RNA-sequencing reads [3]. This tool has been widely adopted in many genome projects to improve the genome assembly and gene annotation [5–8]. Vij et al. improved the contiguity of a 90× coverage PacBio sea bass genome assembly using L_RNA_scaffolder with an assembled transcriptome [9]. In contrast to a strategy using long single-end transcripts, Mortazavi et al. applied paired-end RNA-sequencing reads into scaffolding with RNAPATH [10]. BESST_RNA (https://github.com/ksahlin/BESST_RNA), Rascaf [11], and AGOUTI [12] are other scaffolders using paired-end RNA-sequencing data. However, these tools are either error-prone, or have complicated processes and long runtimes. In addition, studying the influences of RNA-sequencing depth and breadth on scaffolding performance will improve the scaffolding of genomes using RNA-sequencing reads.

In the present study, we developed a fast and accurate tool, called P_RNA_scaffolder, to scaffold genomes using paired-end RNA-sequencing reads. Compared to other similar scaffolders using RNA-sequencing reads, this tool produces the most connections with the shortest runtime and the highest accuracy. One notable advantage of this tool is that the improved proportions of fully covered protein-coding and non-coding genes after scaffolding are close to the proportions in the finished genome. We also examined the practicability of P_RNA_scaffolder for improving the genome assemblies generated by current mate-pair scaffolding and third-generation sequencing assembly.

## Methods

### Algorithm of the P_RNA_scaffolder

High-quality reads obtained with SolexaQA++ [13] were provided to P_RNA_scaffolder for scaffolding. To support paired-end RNA-sequencing reads as evidence, we modified the scaffolding algorithm of L_RNA_scaffolder, which adopted the strategy of maximal supporting evidence to connect genomic sequences [3]. This previous scaffolder only supports single-end RNA-sequencing reads as scaffolding evidence. The modifications in the present study are summarized as follows:

1. First round of alignment and filtration

We align RNA-sequencing reads to contigs with short-read mappers. In eukaryotes mature RNAs are spliced, so we align paired-end RNA-sequencing reads using HISAT2 [14], a fast and sensitive spliced alignment tool. Since mRNA is not typically spliced in prokaryotes [15], BWA-mem algorithm in the BWA package [16] is used to align RNA-sequencing reads to prokaryotic genomes. To rapidly align reads to contigs, multiple processors are utilized. We retain those pairs for which two reads are uniquely aligned to two different contigs.

(2) Second round of alignment and re-filtration

To filter out pairs for which both ends are located on one contig, the retained pairs in the first step are further realigned to contigs using BLAT [17]. If two reads of one pair are realigned to the same contig, or if at least one read is realigned to multiple contigs over a certain minimal length coverage (MLC), then this pair is discarded. The final retained pairs are selected as ‘guides’. One ‘guide’ pair corresponds to two different contigs. This pair is considered the supporting evidence to connect two corresponding contigs. The pair number aligned to these two contigs was considered as the weight of this connection.

(3) Ordering the contigs

Since one ‘guide’ pair is located at two different contigs, two corresponding contigs are ordered and oriented based on the orders of two reads in this pair. In this connection, the first contig is considered as the donor and the second sequence is the acceptor.

(4) Filtering erroneous connections of large inserted size

It is assumed that the alignment regions (*a* and *b*) corresponding to two reads are located in donor *A* and acceptor *B*, respectively. The median exon length was approximately 170 nt across metazoans [18], smaller than the total length of the two reads in one pair. Exons larger than 300 nt were infrequent exceptions [19]. Therefore, two reads in one pair are likely respectively located at two exons. The inserted DNA sequence between *a* and *b* is an intron. The minimal size of the inserted size is equal to [Length(*A*) - end(*a*) + start(*b*)]. Length (A) is the length of donor *A*, end (*a*) is the end position of region *a* in donor *A* and *start (b)* is the start position of region *b* in acceptor *B*. If the minimal size of an inserted DNA length is smaller than a certain maximal intron length (MIL), then the connection is reliable. Otherwise, this connection is filtered out.

(5) Optimizing connections and forming scaffolding graphs

The number of RNA-sequencing pair supporting the connection was the sequencing depth of this region and considered the connection weight. Among all retained connections, one contig could be a donor and/or an acceptor in different connections. For each donor, the connection with the most weight (maximal supporting pairs) is selected. If many connections had the most weight, then they might result from repetitive regions or misalignments and were filtered out. Similarly, the connection of each acceptor is optimized. Then, we generate the scaffolding graphs by linking all optimal connections following a previous strategy [3].

(6) Estimating gap size

To estimate the gap size of two connected contigs, we select all pairs for which two reads are aligned to the same contig. The region between two alignments in a contig is an intron. We estimate the median intron size from all selected alignments. If the MIL of one connection is smaller than the median size, then we insert a sequence comprising the letter ‘N’, the number of which is the difference between two sizes. Otherwise, we insert 100 Ns.

### Human RNA-sequencing reads and genome assembly for parameter simulation

To study the influence of scaffolding parameters and estimate the performance, 36,437 high-quality contigs in hg38 assembly (N50 size of 148.7 kb, downloaded from NCBI GenBank [20]) were used for scaffolding performance comparison. We did not select the sequences generated by de novo assemblers for comparison, as these de novo tools produced mis-assemblies [21], which might interfere with the accuracy estimation of P_RNA_scaffolder. The paired-end RNA-sequencing reads from brain, liver, lung and cells used in the present study are listed in Additional file 1: Table S1. We selected the libraries prepared with the Ribosomal Depletion Kit. This strategy can capture RNA-sequencing reads from both non-polyA RNAs and polyA RNAs [4] and cover protein-coding and non-coding gene regions.

### Estimating scaffolding accuracy and performance

We measured the accuracy of P_RNA_scaffolder following the Genome Assembly Gold Standard Evaluations pipeline [21]. Assuming that the reference assembly is completely correct, we compared the connections by P_RNA_scaffolder with those in the reference assembly and tallied all connections into six types. (i) Consistency: two scaffolded contigs have the same order and orientation as the reference assembly. (ii) Correctable relocations: two contigs with a distance smaller than MIL in one reference chromosome are linked together by P_RNA_scaffolder. (iii) Inversion: Compared with the reference assembly, two scaffolded contigs have the same order but opposite orientations. (iv) Erroneous relocations: two contigs with a distance longer than MIL in one reference chromosome are linked together by P_RNA_scaffolder. (v) Translocations: two scaffolded contigs are distributed at two reference chromosomes. (vi) Unknown: two scaffolded contigs are distributed at two scaffolds of the reference assembly and the accuracy could be not assessed. The links of (i) and (ii) were considered correct. The scaffolding correctness was calculated as the ratio of (correct connections / total connections).

The N50 size was calculated to be a scaffolding performance indicator without consideration scaffolding errors. To produce accurate scaffolding graphs, we also measured the corrected N50 size after splitting scaffolds at every error point (including inversions, erroneous relocations and translocations).

### The scaffolding parameter simulation to balance N50 size and accuracy

The scaffolding performance and accuracy of P_RNA_scaffolder is influenced by supporting pair number, MLC, and MIL. We varied the parameters and compared the resulting N50 size and accuracy. First, to study the influence of supporting pair number on scaffolding performance, MLC and MIL were set as 0.9 and 100 kb, respectively. Then, we calculated the scaffolding performance and accuracy with the pair number from one to five. Notably, the more stringent the supporting guide pair number, the fewer pairs that are used to scaffold, leading to a smaller N50 size. In contrast, because the more stringent guide pair number would filter out the sequencing error or translocation, the more stringent the supporting guide pair number, the higher scaffolding accuracy. Second, with the supporting pair number of two and the MIL of 100 kb, we calculated the scaffolding performance and accuracy when the MLC increased from 0.5 to 0.99. Third, with the supporting pair number of two and MLC of 0.9, we set the MIL from 10 kb to 500 kb and examined the change of the scaffolding performance and accuracy at each MIL.

### Calculating genome coverage in different sequencing depths and breadths

The genome coverage of aligned RNA-sequencing reads affects the scaffolding performance. It is reasonable that the scaffolding performance increases with increasing genome region sequenced. Genome coverage is measured as the ratio of the total bases covered in the alignment regions to all genomic bases [22]. We aligned RNA-sequencing reads to the human hg38 assembly using HISAT2 [14] and selected the pairs of which two ends were aligned to the same chromosome. The genomic regions between the alignment regions of two ends were covered by this pair. To investigate the effect of genome coverage on scaffolding performance, we calculated the correlation coefficient (*R*) between N50 size improvement and genome coverage and investigated whether the *R* was statistically significant using Student’s *t-*test. We also calculated the *R* between corrected N50 size improvement and genome coverage and the significance of the *R*.

Genome coverage by RNA-sequencing reads is affected by sequencing depth and breadth. The sequencing depth was measured as the RNA-sequencing pair number. To assess the effect of sequencing depth to genome coverage and performance, we sampled ten subsets from 10% to 100% of brain RNA-sequencing reads and then calculated genome coverage, the scaffolding performance and accuracy of each subset.

The sequencing breadth comprises sequenced tissues and developmental stages. Because of the spatial and temporal expression features of genes, increased sequencing breadth, including multiple tissues and developmental stages, would cover more genomic regions. To investigate the influence of sequencing breadth on performance, we constructed six RNA-sequencing datasets from four tissues and scaffolded the contigs using each dataset. The genome coverage, scaffolding performance and accuracy of each subset were calculated.

### Estimating the proportion of fully covered protein-coding genes and non-coding RNAs

The proportions of fully covered proteins and non-coding RNAs were used to measure the gene completeness in different assemblies. To evaluate the proportions of fully covered protein-coding genes, with BLAT [17] we aligned human Swiss-Prot [23] proteins to three assemblies (the contigs, the P_RNA_scaffolder assembly and the hg38 assembly). If one protein had an alignment where the sequence coverage was over 0.9, then this protein was fully covered. Next, we calculated the proportion of fully covered proteins in all proteins.

To evaluate the proportions of fully covered non-coding genes, human circular RNAs (circRNAs) downloaded from circBase [24] were aligned to three assemblies with SPALN [25]. If the alignment coverage of one circRNA was over 0.9, then it was considered complete in the assembly. Then, we calculated the proportion of complete circRNAs in all circRNAs.

### Comparison of the performances of P_RNA_scaffolder and other tools using various species

Five tools using paired-end RNA-sequencing reads for genome scaffolding were used, including RNAPATH [10], L_RNA_scaffolder [3], Rascaf [11], AGOUTI [12] and BESST_RNA (https://github.com/ksahlin/BESST_RNA). RNAPATH with the denoised joining-pairs, which were produced by AGOUTI, had better performance than itself without denoising steps [12]. Thus, in the comparison, we ran RNAPATH with the denoised joining-pairs. We compared the present method with these five tools in human, *C. elegans* and *E. coli*, which corresponded to genomes of large, medium and small sizes, respectively. For human, the brain RNA-sequencing reads were used to scaffolding 36,437 high-quality contigs in hg38 assembly. For *E. coli* (K-12 strain, MG1655 substrain), the reference assembly was obtained from the NCBI Genome database. Since the high-quality reference assembly is finished and consists of only a sequence, we randomly fragmented the whole assembly into 100 contigs (N50 size of 76 kb). A total of 38,915,405 cleaned RNA-sequencing pairs were used to scaffold (Accession in NCBI SRA database: SRR1931786). For *C. elegans* (WBcel235), 21,965,772 cleaned RNA-sequencing pairs (Accession in NCBI SRA database: SRR4017997) were used as the input of six tools. Similar to *E. coli*, the finished reference assembly of *C. elegans* is of such high quality that only six chromosome sequences are included. To re-scaffold the *C. elegans* genome for comparison, 2000 contigs (N50 size of 84.4 kb) were generated by randomly splitting the reference assembly. Each scaffolder was run with a supporting pair number of two. The alignments of RNA-sequencing reads to contigs and each scaffolding result were run with twenty threads on the same machine.

### Data used in the present study and scaffolder usage

All data used in the present study are available in NCBI SRA database, as stated in Additional file 1: Table S1. P_RNA_scaffolder together with the RNA-sequencing reads in the present study, are available at http://www.fishbrowser.org/software/P_RNA_scaffolder.

## Results

### Balancing N50 size and accuracy by different parameters

**Fig. 1:**
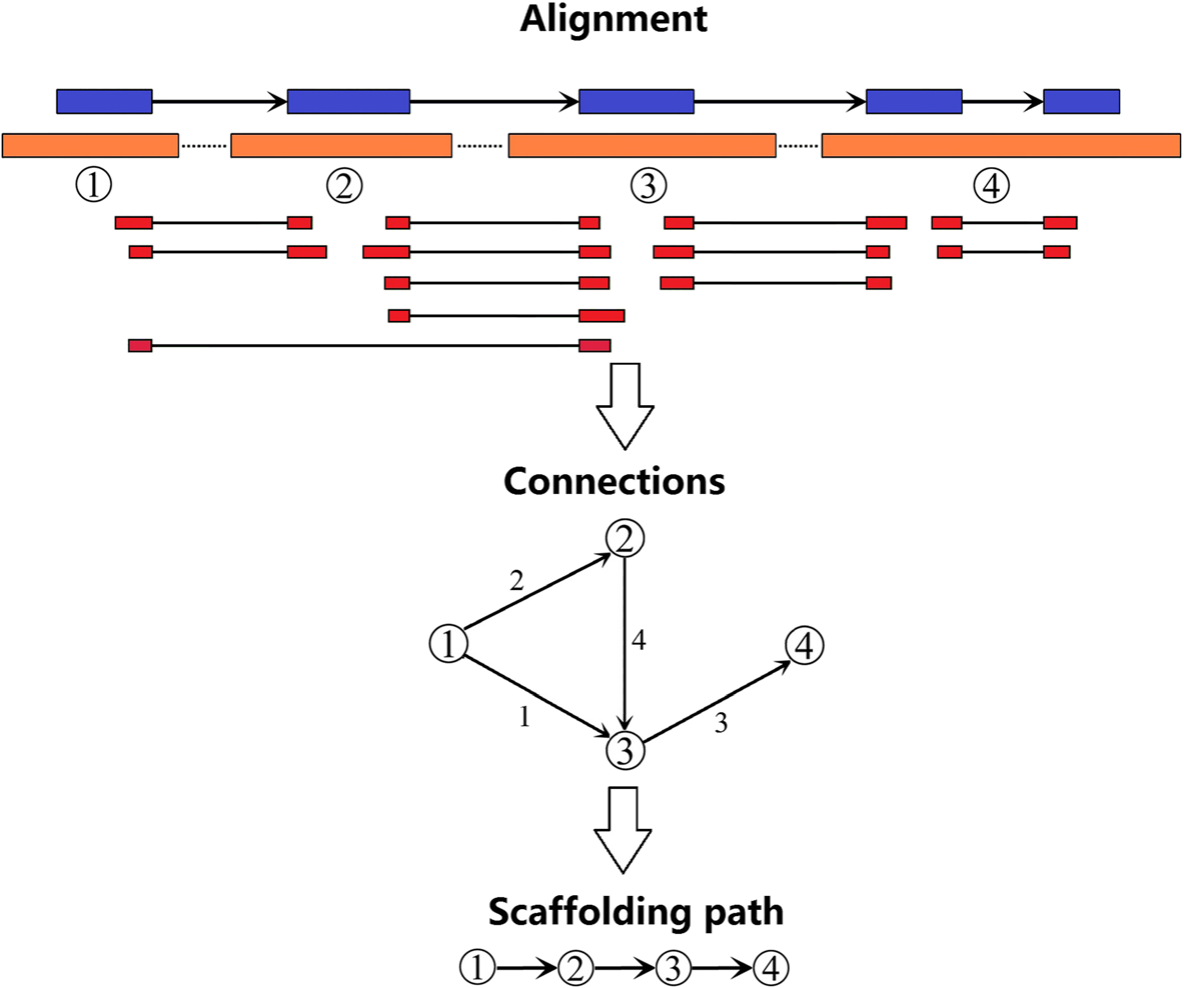
The main steps of P_RNA_scaffolder. Main steps of P_RNA_scaffolder. **i)** Paired-end reads (red) generated from transcripts (blue) are aligned to genomic fragments (orange). The arrows represent transcript orientation. We retained those pairs for which two ends are aligned to two contigs. **ii)** The pair is the connection evidence of two contigs. For each connection, we calculated the supporting evidence numbers of all connections. In one connection, two contigs are ordered based on their positions in the pairs and classified into the start and the end. **iii)** We select the optimal connection for each start contig and each end contig. The retained optimal connections are attributed into scaffolding paths

Compared with long single-end RNA-sequencing technology, the simplicity, cheapness, and high throughput of paired-end RNA-sequencing technology could make RNA-sequencing widely applicable for genome scaffolding. However, scaffolding strategies using paired-end RNA-sequencing reads are distinct from L_RNA_scaffolder using single-end RNA-sequencing data. Although the method could employ assembled transcripts from paired-end RNA sequencing reads, this strategy required transcriptome de novo assembly, leading to much long runtime and low accuracy. Therefore, to be compatible with paired-end RNA sequencing reads, we modified the core algorithm of a previous scaffolder. Compared with the previous tool, this new method has two important modifications. First, in L_RNA_scaffolder, we aligned long single-end transcripts using BLAT [17], which was suitable for the accurate alignment of low-throughput sequences. Different from the strategy to align long transcripts, short-read mappers, such as HISAT2 [14] and BWA [26], were supported in the new tool. Second, the paired-end RNA-sequencing reads were shorter than the long single-end reads. One pair might be aligned to two contigs at most, while a long single-end read might be covered in multiple contigs. Hence, one pair could support only one connection but one single-end read could support multiple connections. The core algorithm of P_RNA_scaffolder is outlined in Fig. 1 and Additional file 2: Figure S1.

**Fig. 2:**
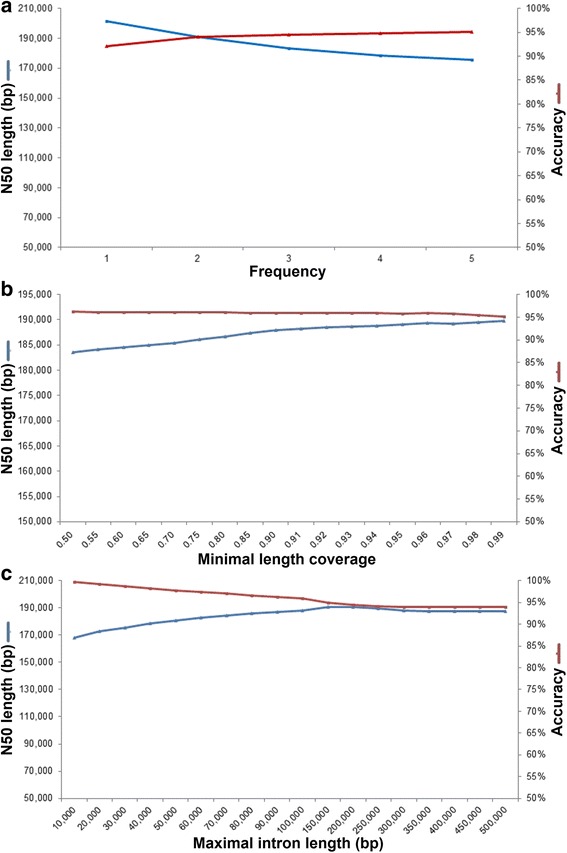
Influence of supporting pair number, MLC and MIL on the performance of P_RNA_scaffolder. **a** Influence of supporting pair number on the performance and accuracy of P_RNA_scaffolder. As the supporting pair number increased, the accuracy (red line) increased but the N50 size (blue line) decreased. **b** Influence of MLC on the performance of P_RNA_scaffolder. The N50 size (blue line) increased but the accuracy (red line) decreased with increasing MLC. **c** Influence of MIL on the performance of P_RNA_scaffolder. The accuracy (red line) exhibited the opposite trend to the N50 size (blue line)

To assess the accuracy and performance of P_RNA_scaffolder, we used the human genome as a reference because this genome assembly was almost complete and well-collected paired-end RNA-sequencing data were available. We built scaffolds using P_RNA_scaffolder from 36,437 initial contigs in hg38 assembly (assembly size of 3.09 Gb with N50 size of 148.7 kb). We studied the effects of supporting pair number, minimal length coverage (MLC), and maximal intron length (MIL) to the performance and accuracy with brain RNA-sequencing reads. When MLC and MIL were set as 0.9 and 100 kb, respectively, the N50 size deceased as the supporting pair number increased. When the supporting pair number was one (Fig. 2a), the accuracy was 94.03%, with a resulting N50 size of 201 kb. If the pair number was over two, then the accuracy was higher than 96%, but the resulting N50 size was smaller than 188.4 kb. The supporting pair number exhibited a negative correlation with the N50 size but a positive correlation with the accuracy. For MLC simulation where the MIL and supporting pair number were set as 100 kb and two, the N50 size increased over 188.4 kb but the accuracy decreased to 96% as MLC increased to more than 0.9 (Fig. 2b). For MIL simulation with the supporting pair number of two and MLC of 0.9, the accuracy decreased from 99.69% to 96% accompanying the increase of N50 size when the MIL was more than 100 kb (Fig. 2c). The MLC and MIL exhibited a positive correlation with N50 size but a negative correlation with accuracy. These three simulations revealed the opposite effects of supporting pair number, MLC and MIL to scaffolding performance and accuracy.

### Improving the performance by multiple RNA-seq datasets from increasing sequencing depth and breadth

**Fig. 3:**
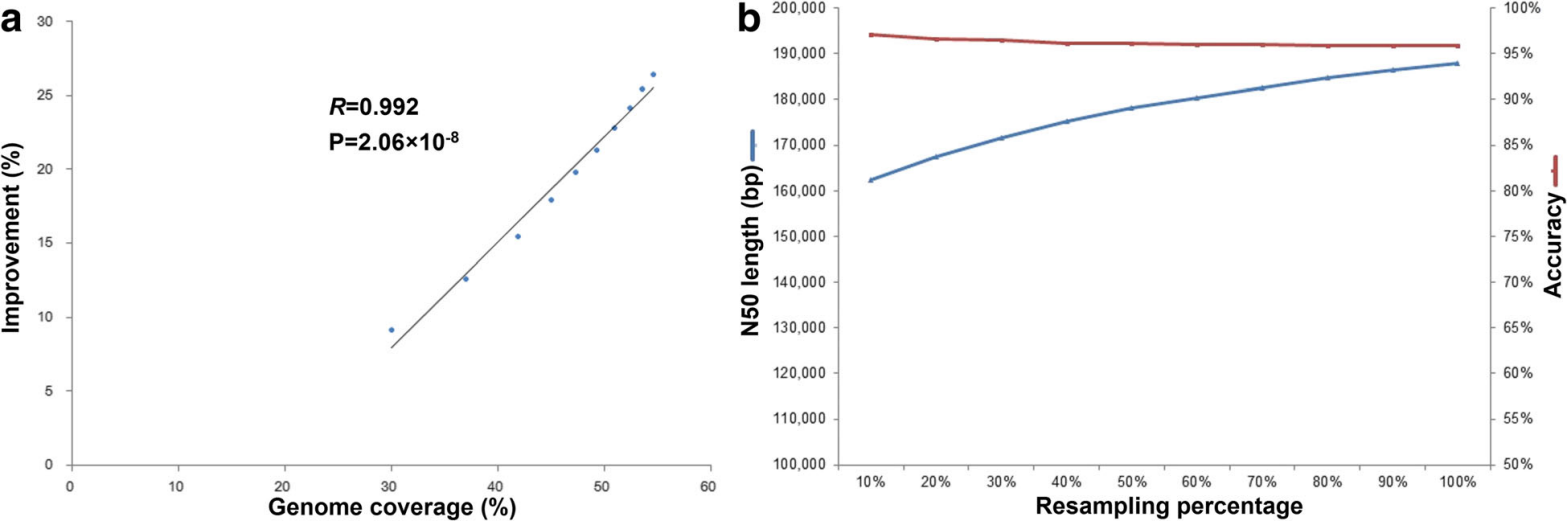
The influence of sequence depth on performance. **a** Using ten scaffolding results generated by sampling brain RNA-sequencing reads, we found significant correlation between N50 size improvement and genome coverage. *R* was the correlation coefficient. *P* was the statistical value of Student’s *t*-test for *R*. **b** The N50 size (blue line) increased with the increased sequencing depth but is not saturated. During the sampling, the accuracy (red line) was approximately 96%

To study the influence of sequence depth and breadth on scaffolding, the optimal supporting pair number, MLC and MIL parameters were set as two, 0.9 and 100 kb, respectively. The optimal parameters were used to balance the N50 size and accuracy. We sampled human brain RNA-sequencing reads from 10% to 100%. The corresponding genome coverage increased from 30.01% to 54.60% and the N50 size reached 188.4 kb (26.71% increase compared to that in the initial contig, Additional file 1: Table S2). A significant correlation was observed between genome coverage and N50 size (correlation coefficient *R* = 0.992, Student’s *t*-test *P* value = 2.06 × 10^− 8^, Fig. 3a). If we corrected the scaffolds by splitting scaffolds at every error point, then the N50 size was also significantly correlated with genome coverage (*R* = 0.993, Student’s *t*-test *P* value = 8.16 × 10^− 9^, Additional file 2: Figure S2a). We inferred that the N50 was not saturated and would increase as more transcriptome data became available (Fig. 3b).

**Fig. 4:**
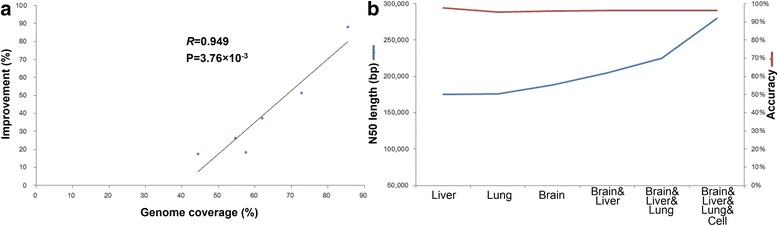
The influence of sequence breadth on performance using different tissue sets. **a** With increasing sequence breadth, we observed significant correlation between N50 size improvement and genome coverage. **b** The N50 size (blue line) increased with increasing number of samples but was not saturated. In all samples the accuracy (red line) was almost not changed (~ 96%)

To assess the effect of sequence breadth to scaffolding performance, we constructed six datasets of RNA-sequencing reads from different tissues, including (i) brain, (ii) liver, (iii) lung, (iv) brain and liver, (v) brain, liver and lung, (vi) brain, liver, lung and cells. With enlarged RNA-sequencing reads from different datasets, the genome coverage increased from 44.42% to 85.5% (Additional file 1: Table S3). With the parameters (supporting pair number of two, MLC of 0.9, and MIL of 100 kb), the N50 size was improved with increasing number of sequenced tissues; a significant correlation was observed (*R* = 0.949, Student’s *t*-test *P* value = 3.76 × 10^− 3^, Fig. 4a). The corrected N50 size was also significantly correlated with genome coverage (*R* = 0.938, Student’s *t*-test *P* value = 5.55 × 10^− 3^, Additional file 2: Figure S2b). With all RNA-sequencing reads from four tissues (brain, liver, lung and cells), P_RNA_scaffolder generated 10,849 connections, covering a total of 2.74 Gb (85.5%) of human genome. The final N50 size was 279.9 kb (an 88.2% increase compared to that in the initial contig set, Additional file 1: Table S3) with an accuracy of 96.14%. The corrected N50 size was 262.5 kb, showing an improvement of 76.54%. Even with all studied reads, the N50 size was not saturated (Fig. 4b). Taken together, these results indicated that enlarged sequencing depth and breadth could increase the scaffolding performance.

### The completeness of protein-coding gene regions and non-coding gene regions is improved and close to those in human genome

**Fig. 5:**
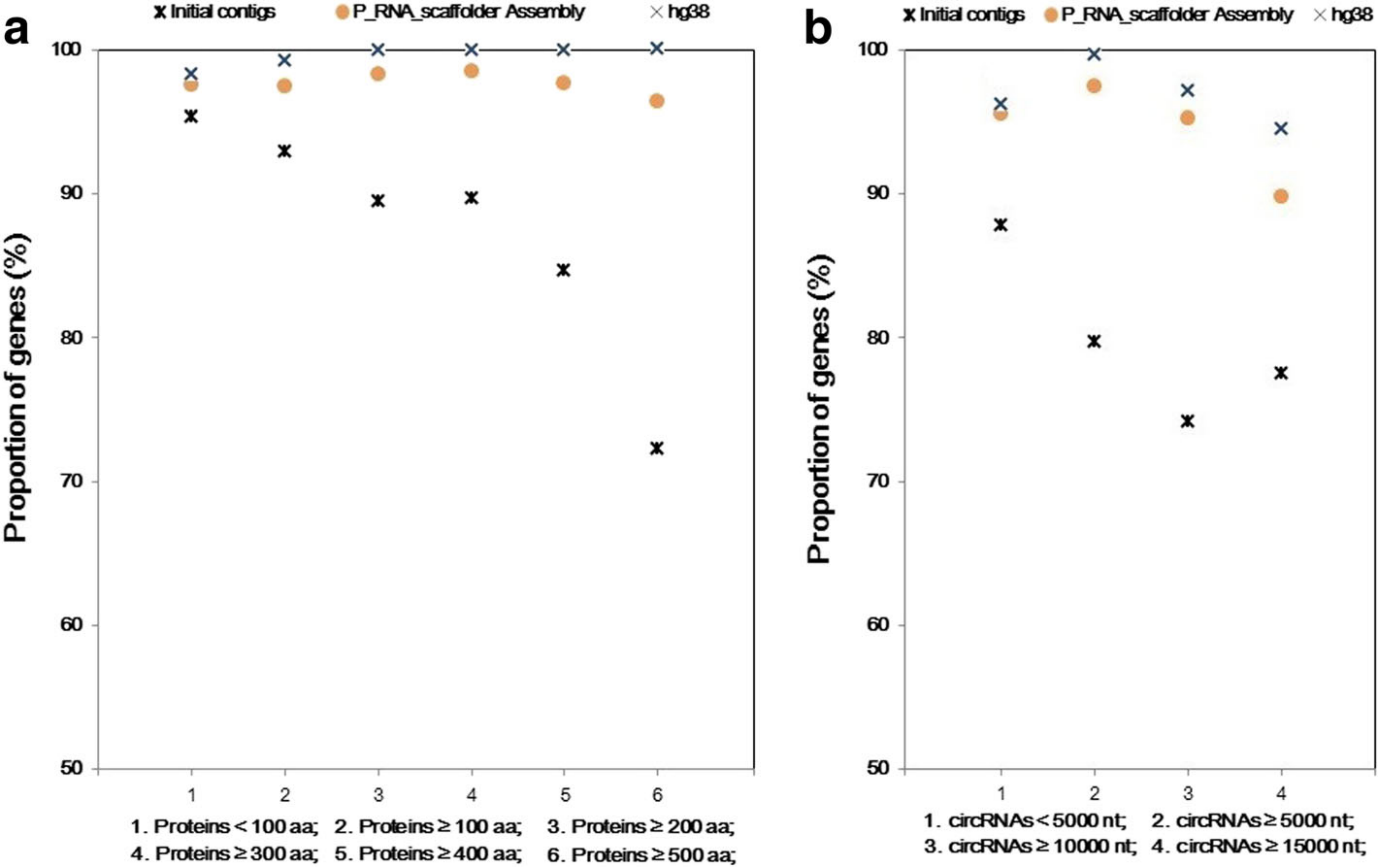
The proportions of fully-covered genes in three human assemblies. **a** The coverage of Swiss-Prot proteins; and **b** the coverage of circRNAs

To assess the coverage improvement of protein-coding genes after scaffolding, we aligned 20,208 human Swiss-Prot proteins to three assemblies (the contigs, P_RNA_scaffolder assembly and hg38 assembly) using BLAT [17]. With a length coverage threshold of 90%, the proportions of fully covered proteins were 82.8% (in the contigs), 97.4% (in the P_RNA_scaffolder assembly) and 99.8% (in hg38 assembly). Specifically, the percentage of recovered proteins over 500 amino acids showed a larger increase (from 72.2% to 96.4%) than that of shorter proteins (Fig. 5a).

We also examined the coverage improvement of non-coding genes after scaffolding. A total of 140,790 human circRNAs from circBase [24] were aligned to three assemblies with SPALN [25]. The percentage of fully covered circRNAs increased to 95.6% in the P_RNA_scaffolder assembly, higher than that in the contigs (87.4%) and close to that in hg38 (96.2%). Particularly, the percentage of complete circRNAs over 5000 bp showed a larger improvement (from 78.4% to 96.2%) compared to that of shorter circRNAs (Fig. 5b). These results showed that the present method significantly recovered the structures of both protein-coding genes and non-coding genes.

### P_RNA_scaffolder is faster and more efficient than other tools among various species

**Fig. 6:**
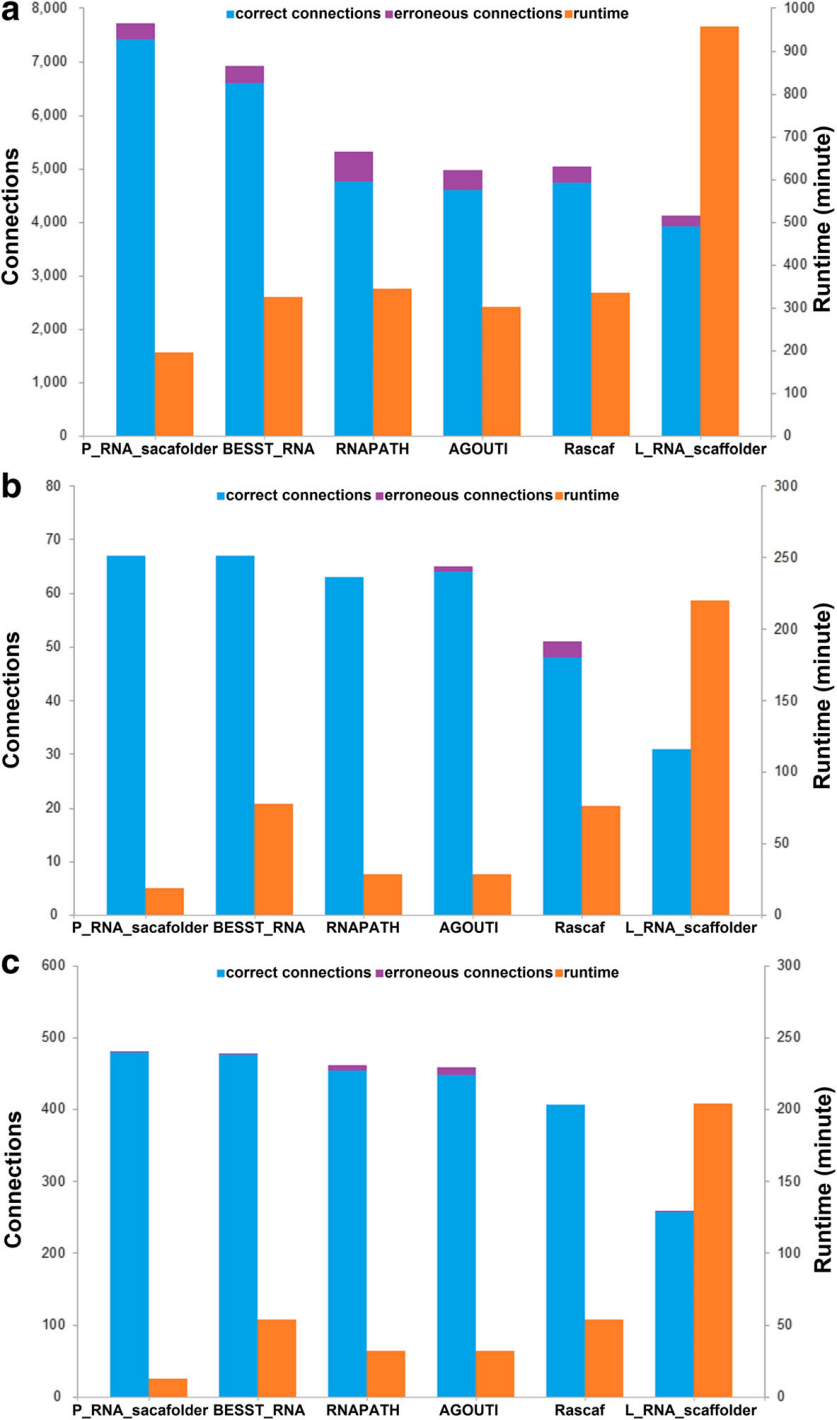
The scaffolding connection number, accuracy and runtime of six tools using RNA-sequencing reads. **a** Comparison in human. **b** Comparison in *E. coli*. **c** Comparison in *C. elegans*

To compare the performance of the present method with that of five existing tools of the same type (RNAPATH [10], L_RNA_scaffolder [3], Rascaf [11], AGOUTI [12] and BESST_RNA), we used these six tools to scaffold the contig sets from the reference assemblies of human, *C. elegans* and *E. coli*. Assuming that the contig sets had no mis-assemblies, we computed the connection number, accuracy, and runtime of each tool. Using human brain RNA-sequencing reads, among the six scaffolding results of human contigs, P_RNA_scaffolder produced the most connections (7727, Fig. 6a; Additional file 1: Table S4). The accuracy of the present method (96%) was also higher than that of the other five scaffolders. P_RNA_scaffolder had a runtime of 195 min, which was approximately two-thirds of the runtime of AGOUTI (the second fastest tool, 301.35 min and 92.47% accuracy, Additional file 1: Table S4 and Table S5).

We also compared the performance of each scaffolder on small (*E. coli*) and medium size (*C. elegans*) size genomes. In *E. coli*, the present tool and BESST produced more connections (67) than the other tools (65 by AGOUTI, 63 by RNAPATH, 51 by Rascaf, and 31 by L_RNA_scaffolder, Fig. 6b, Additional file 1: Table S6). The scaffolding accuracy of P_RNA_scaffolder reached 100%, equal to that of BESST_RNA, RNAPATH and L_RNA_scaffolder, but higher than that of AGOUTI (98.46%) and Rascaf (94.12%). However, the runtime of P_RNA_scaffolder was the shortest (19 min) and only two-thirds the runtime of AGOUTI, the second fastest tool (Additional file 1: Table S7). In *C. elegans*, the present tool produced more connections (480) than the other tools (478 by BESST_RNA, 461 by RNAPATH, 458 by AGOUTI, 407 by Rascaf, and 259 by L_RNA_scaffolder, Fig. 6c, Additional file 1: Table S8) with a shorter runtime (12.77 min) than the other tools (Additional file 1: Table S9). Additionally, P_RNA_scaffolder produced only one mis-assembly, with the second highest scaffolding accuracy (99.79%) among all tools. Taken together, comparison of the performances of P_RNA_scaffolder and other tools among various species demonstrated that P_RNA_scaffolder outperformed the existing state-of-the-art scaffolders using paired-end RNA-sequencing reads.

### Our tool improves the contiguity of genome assembly generated by the mate-pair scaffolding strategy

Mate-pair based scaffolding is dominant in genome projects. Mate-pair libraries are widely used for genome scaffolding. In a previous study [3], four mate-pair libraries of distinct insert sizes (2, 5, 10 and 35 kb) with the same amount of pairs (8.8 million) were employed into scaffolding human contigs using five scaffolding tools (Opera [27], Soapdenovo [28], MIP scaffolder [29], SSPACE [30], and SOPRA [31]). A total of 19 scaffolding results were generated, except for using SOPRA with the library of 35 kb inserts. We compared the present method with those of mate-pair scaffolding tools with respect to performance. The input RNA-sequencing read number was equal to that of mate-pair reads in all four libraries (8.8 million pairs). P_RNA_scaffolder generated an assembly with the N50 size of 277 kb and an accuracy of 97%. The N50 size of P_RNA_scaffolder result was larger than 14 scaffolding results using libraries of 2 kb, 5 kb or 10 kb inserts but smaller than those using the 35 kb library (Additional file 2: Figure S3). The N50 size produced by MIP scaffolder with the 10 kb library was also larger than that of P_RNA_scaffolder. The accuracy of P_RNA_scaffolder (97%) was higher than that of MIP scaffolder using the 35 kb library (82.39%) and Opera using the 10 kb library (86.21%), and close to the results of the other 17 scaffolding results (≥98.45%).

**Fig. 7:**
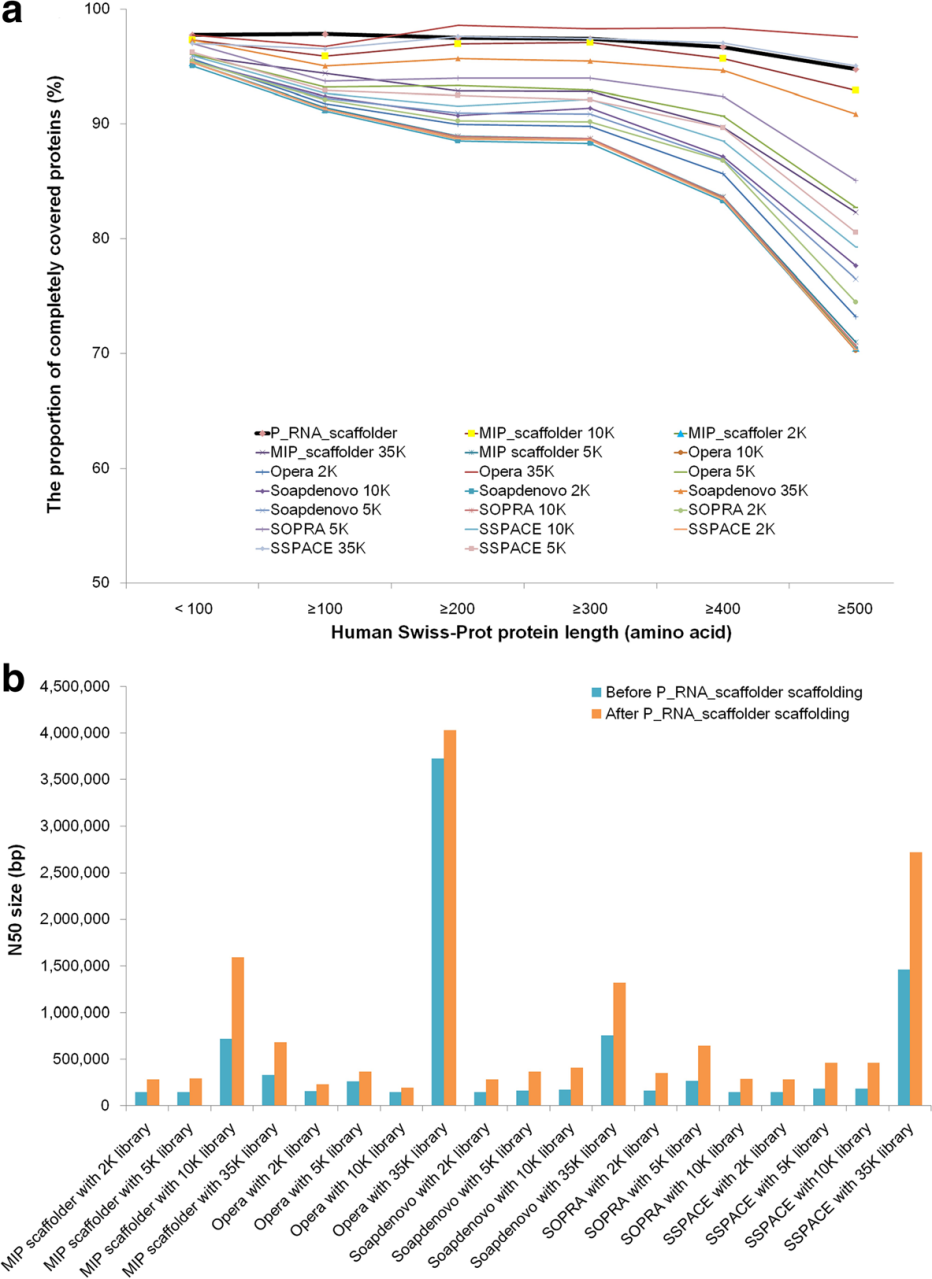
Comparison of P_RNA_scaffolder and five scaffolders using mate-pair libraries. **a** Swiss-Prot proteins were aligned to 20 scaffolding results from the human genome using BLAT. The parameters were the same as those in Fig. 5a. The proportion of completely covered protein-coding genes in P_RNA_scaffolder assembly ranked second (black line). **b** The improvements of 19 assemblies by using P_RNA_scaffolder

P_RNA_scaffolder is complementary to current mate-pair scaffolding strategies. First, assembly by the present tool exhibited considerably higher completeness of gene regions than most assemblies by a mate-paired strategy. To assess the improvement of gene coverage with the different methods, we aligned human Swiss-Prot proteins to each scaffolding assembly using BLAT. As shown in Fig. 7a, with a length coverage threshold of 90%, the proportion of completely covered protein-coding genes in P_RNA_scaffolder (96.35%) was higher than those in 18 scaffolding results but only less than that in the assembly produced by OPERA with the 35 kb library (97.81%). Second, mate-pair approaches are limited by cloning or ligation efficiency and are more costly [32, 33]. The simplicity and high throughput of RNA-sequencing technology could make transcriptome reads widely applicable to genome scaffolding. Third, a hybrid strategy using first mate-pair scaffolding and then P_RNA_scaffolder will significantly improve the contiguity of assembly. Using P_RNA_scaffolder, we further scaffolded these 19 assemblies. The N50 sizes of the new assemblies were 1.08~ 2.54-fold higher than the 19 assemblies prior to scaffolding (Fig. 7b and Additional file 1: Table S10). Taken together, these data demonstrated that P_RNA_scaffolder provided not only a practical alternative to the existing scaffolding methods for N50 improvement but also a better solution to improve gene coverage.

### Our tool improves the contiguity of genome assembly generated by the third-generation sequencing assembly strategy

The third-generation of single-molecule sequencing strategy has been used for genome assembly, and can produce much longer contigs [34, 35]. Our previous tool, L_RNA_scaffolder, had been demonstrated to improve the contiguity of long-read (Pacific Biosciences, or PacBio) genome assembly [9]. We also examined the improvement of the PacBio genome assembly by P_RNA_scaffolder. Berlin et al. generated de novo assemblies of *Saccharomyces cerevisiae* and human hydatidiform mole cell line (CHM1) with SMRT sequencing reads [36]. The assembly contig N50 sizes of *S. cerevisiae* and human CHM1 were 818,518 bp and 5,082,961 bp, respectively. To scaffold the PacBio assembly of *S. cerevisiae*, 30,485,207 pairs of cleaned RNA-seq (Accessions in NCBI SRA database: SRR5417301 and SRR5417312) were inputted into P_RNA_scaffolder and the final N50 size was 946,396 bp with a 15.6% increase. With human RNA-seq reads from brain, lung, liver, and cells, the N50 size of human Pacbio assembly was improved to 6,889,382 bp (a 35.5% increase). These two applications of P_RNA_scaffolder to PacBio assemblies demonstrated that the present tool was also complementary to third-generation single-molecule sequence assembly.

### RNA-sequencing reads of close species is not suitable for scaffolding target genome

Homologous proteins of close species can be used to scaffold target genomes [2, 37]. Thus, we examined whether paired-end RNA-sequencing reads of close species could be employed to scaffold target genomes. Cleaned mouse RNA-sequencing reads (38 million of pairs, Accession in NCBI SRA database: SRR2878547) were used to scaffold human contigs. Only 85 connections were generated with an N50 size improvement of 0.21%. The accuracy was as low as 62.35% (Additional file 1: Table S11). These results indicated few connections and high error rates using RNA-sequencing reads from close species to scaffold a target genome. The major reason for this finding is likely nucleotide variants between species.

## Discussion

Compared with other tools (except the unpublished BESST_RNA), P_RNA_scaffolder exhibited three novelties to ensure its accuracy and efficiency. First, to achieve high accuracy, P_RNA_scaffolder performed two rounds of alignments and filtrations to select uniquely aligned RNA-seq reads whereas other tools performed only one round of alignment and filtration. As expected, an additional round of alignment and filtration improved accuracy compared with the results from only one round of alignment (Additional file 1: Table S12). Second, the present method has fewer prerequisites than other tools. AGOUTI required predicted gene models [12]. Zhang et al. showed that RNAPATH with denoised joining-pairs, produced by AGOUTI, had better performance than itself without denoising steps [12]. Therefore, to obtain better performance and higher accuracy, RNAPATH is also dependent on gene models. However, this requirement of gene models limited their application to only scaffolding the coding genomic regions. The present strategy does not depend on predicted gene models and thus could be applied to scaffolding non-coding gene regions. Rascaf built exon blocks and gene blocks and then detected the order of the gene blocks [11]. De novo transcriptome assembly was time-consuming but necessary for L_RNA_scaffolder [3]. Neither constructing gene blocks nor transcriptome assembly are needed by P_RNA_scaffolder. Third, the speed and accuracy of determining the connections is another challenge. AGOUTI and RNAPATH determined reliable connections by employing RNA-seq alignments and predicted gene models. After splitting scaffolds into contigs, Rascaf incorporated RNA-sequencing alignments and initial scaffold connections to form new contig graphs. P_RNA_scaffolder directly utilized the RNA-seq alignments as evidence and adopted the strategy of maximal support evidence for scaffolding. In various organisms, P_RNA_scaffolder outperformed existing state-of-the-art scaffolders, generating the most connections with the shortest runtime and highest accuracy. These comparisons demonstrate that the present strategy to determine accuracy connections is faster and more efficient than the combination of RNA-sequencing alignments and additional evidence.

Alternative splicing may influence the accuracy of P_RNA_scaffolder. We carefully examined three potential cases where alternative splicing exists in the scaffolds. (1) If all isoforms were distributed at the same genome sequences, then the reconstructed scaffold could completely cover all alternative splicing isoforms (Additional file 2: Figure S4a). (2) If one isoform includes all exons and has dominant expression, then P_RNA_scaffolder uses the reads of this isoform to construct the connecting paths (Additional file 2: Figure S4b). The scaffold covers all alternative splicing isoforms. (3) If the dominantly expressed isoform consists of a part of exons, then the alternative exonic contigs are not scaffolded, leading to a relocation event (Additional file 2: Figure S4c). Some isoforms could not be completely recovered. To evaluate the influence of alternative splicing events on the accuracy of the present method, 161,844 human splicing isoforms (to 20,272 genes) annotated by Ensembl database [38] were used as a test dataset. Among these genes, 161,199 isoforms (99.6%) were completely aligned to P_RNA_scaffolder result using BLAT. This result indicated that alternative splicing had little influence on the present scaffolding strategy.

Another challenge is that low-abundance RNA-sequencing reads generated from transcriptional noise might influence P_RNA_scaffolder accuracy. Low-abundance transcripts might be active genes. The expression of protein-coding genes measured through RNA-sequencing shows a bimodal distribution of high and low expression [39]. In addition, the expression of long non-coding RNAs is on average lower than that of protein-coding genes [40, 41]. However, the low-abundance RNA-sequencing reads might also result from technical or biological noise, which might lead to errant scaffoldings. We determined that the connection threshold is two. As shown in Fig. 2a, the accuracy was higher than 96% with the pair number (sequencing depth) ≥ two. That is, a region having a sequencing depth ≥ two might represent active transcription rather than transcription noise.

The advantages of the present tool were demonstrated by two different uses. Compared with the 19 scaffolding results using mate-pair libraries, the N50 size of the present tool ranked fifth, but the gene completeness of the present tool ranked second. Since RNA-sequencing technology is simpler and more efficient than mate-pair sequencing, paired-end RNA-sequencing should make transcriptome reads widely applicable to genome scaffolding. Moreover, the present tool could improve the contiguity of genome assembly generated by current mate-pair scaffolding strategy and third-generation single-molecule sequencing assembly strategy. Therefore, P_RNA_scaffolder can make a significant contribution to genome assembly and gene prediction.

## Conclusions

Using paired-end RNA-seq reads, P_RNA_scaffolder can improve the contiguity of genome assembly. It exhibits higher speed and efficiency than the existing state-of-the-art scaffolders. It also provides a fast and accurate alternative to the existing assembly methods. Furthermore, after scaffolding the completeness of protein-coding and non-coding gene regions is improved and close to that in reference genome. The promising outcomes indicate that this tool can be of value and wide use for gene prediction. Overall, P_RNA_scaffolder can improve the contiguity of genome assembly and benefit gene prediction.

## Additional files

Additional file 1: **Tables S1.** The SRA accessions of human paired-end RNA-sequencing reads. **Table S2.** The performance of P_RNA_scaffolder using ten datasets of RNA-sequencing reads from brain. **Table S3.** The performance of P_RNA_scaffolder with paired-end RNA-sequencing data from different tissues. **Table S4.** The accuracy and performance of six scaffolders on human contigs. **Table S5.** The runtime of six scaffolders on human contigs (minutes). **Table S6.** The accuracy and performance of six scaffolders on E.coli contigs. **Table S7.** The runtime of six scaffolders on E.coli contigs (minutes). **Table S8.** The accuracy and performance of six scaffolders on C.elegans contigs. **Table S9.** The runtime of six scaffolders on C.elegans contigs (minutes). **Table S10.** The improvement of genome assemblies from mate-pair scaffolding strategy by P_RNA_scaffoler. **Table S11.** The scaffolding performance of P_RNA_scaffolder with mouse paired-end RNA-sequencing reads on human contigs. **Table S12.** The performance of P_RNA_scaffolder with BLAT alignment and without BLAT alignment. (DOCX 55 kb)

Additional file 2: **Figure S1.** Flow chart of P_RNA_scaffolder. **Figure S2.** The correlation between corrected N50 size improvement and genome coverage. **Figure S3.** Accuracy and N50 sizes of P_RNA_scaffolder and other mate-pair scaffolder. **Figure S4.** Influence of alternative splicing on the accuracy of P_RNA_scaffolder. (DOCX 399 kb)

## Abbreviations

circRNA: circular RNA
MIL: maximal intron length;
MLC: minimal length coverage
