# Correction to: P_RNA_scaffolder: a fast and accurate genome scaffolder using paired-end RNAsequencing reads

**DOI:** 10.1186/s12864-019-5855-2

**Published:** 2019-06-26

**Authors:** Bai-Han Zhu, Jun Xiao, Wei Xue, Gui-Cai Xu, Ming-Yuan Sun, Jiong-Tang Li

**Affiliations:** 1 Key Laboratory of Aquatic Genomics, Ministry of Agriculture, CAFS Key Laboratory of Aquatic Genomics and Beijing Key Laboratory of Fishery Biotechnology, Chinese Academy of Fishery Sciences, Beijing, 100141 China; 2 College of Fisheries and Life Science, Shanghai Ocean University, Shanghai, 201306 China; 3 College of Marine Science, Zhejiang Ocean University, Zhoushan, 316022 China

**Correction to: BMC Genomics (2018) 19:175**

**https://doi.org/10.1186/s12864-018-4567-3**

Following the publication of this article [1], the authors reported that the link to the software described in the article is no longer valid. They have therefore provided the following alternative address in this Correction article in order to gain access to the software: https://github.com/CAFS-bioinformatics/P_RNA_scaffolder
