# Supplementary material for: P_RNA_scaffolder: a fast and accurate genome scaffolder using paired-end RNA-sequencing reads
Source: BMC Genomics. 2018 Mar 2;19:175. doi: 10.1186/s12864-018-4567-3 (PMC5834899; doi:10.1186/s12864-018-4567-3)
Supplement: Supplementary file 1 — Tables S1. The SRA accessions of human paired-end RNA-sequencing reads. Table S2. The performance of P_RNA_scaffolder using ten datasets of RNA-sequencing reads from brain. Table S3. The performance of P_RNA_scaffolder with paired-end RNA-sequencing data from different tissues. Table S4. The accuracy and performance of six scaffolders on human contigs. Table S5. The runtime of six scaffolders on human contigs (minutes). Table S6. The accuracy and performance of six scaffolders on E.coli contigs. Table S7. The runtime of six scaffolders on E.coli contigs (minutes). Table S8. The accuracy and performance of six scaffolders on C.elegans contigs. Table S9. The runtime of six scaffolders on C.elegans contigs (minutes). Table S10. The improvement of genome assemblies from mate-pair scaffolding strategy by P_RNA_scaffoler. Table S11. The scaffolding performance of P_RNA_scaffolder with mouse paired-end RNA-sequencing reads on human contigs. Table S12. The performance of P_RNA_scaffolder with BLAT alignment and without BLAT alignment. (DOCX 55 kb) [file 12864_2018_4567_MOESM1_ESM.docx]

**Supplementary Tables**

**Table S1. The SRA accessions of human paired-end RNA-sequencing reads**

| **Samples** | **Accessions** | | | |
| --- | --- | --- | --- | --- |
| Human brain | ERR420392 | ERR420391 | ERR420386 | ERR420387 |
| Human liver | ERR420389 | ERR420393 | ERR420390 | ERR420388 |
| Human Lung | SRR1297303 | SRR1297304 | SRR1297305 | SRR1297306 |
| Human cells | SRR2166638 | SRR2166637 | SRR2166636 | SRR2166635 |
|  | SRR2166634 | SRR2166633 | SRR2166632 | SRR2166631 |
|  | SRR2166630 | SRR2166629 | SRR2166628 | SRR2166627 |
|  | SRR2166626 | SRR2166625 | SRR2166624 |  |

**Table S2. The performance of P_RNA_scaffolder using ten datasets of RNA-sequencing reads from brain**

| **Sampling percentage** | **10%** | **20%** | **30%** | **40%** | **50%** |
| --- | --- | --- | --- | --- | --- |
| RNA-seq pair number | 11,382,169 | 22,764,338 | 34,146,507 | 45,528,676 | 56,910,845 |
| **Accuracy** | | | | | |
| Consistence | 2,970 | 3,934 | 4,704 | 5,349 | 5,778 |
| Inversions | 3 | 2 | 3 | 3 | 2 |
| Correctable relocations | 188 | 258 | 303 | 368 | 400 |
| Erroneous relocations | 91 | 118 | 147 | 185 | 205 |
| Translocations | 12 | 22 | 30 | 35 | 36 |
| Unknown | 0 | 0 | 0 | 0 | 0 |
| Total correct links^$^ | 3,158 | 4,192 | 5,007 | 5,717 | 6,178 |
| Total links | 3,264 | 4,334 | 5,187 | 5,940 | 6,421 |
| Accuracy | 96.75% | 96.72% | 96.53% | 96.25% | 96.22% |
| **Length** | | | | | |
| N50 length (bp) | 162,340 | 167,147 | 171,084 | 174,991 | 177,969 |
| Improvement | 9.16% | 12.39% | 15.04% | 17.67% | 19.67% |
| Corrected N50 length(bp) | 161,846 | 166,528 | 170,197 | 173,509 | 176,097 |
| Corrected improvement | 8.83% | 11.98% | 14.45% | 16.67% | 18.41% |
| **Sequence Number** | | | | | |
| P_RNA_scaffolder sequence number | 33,173 | 32,103 | 31,250 | 30,497 | 30,016 |
| Initial contigs number | 36,437 | 36,437 | 36,437 | 36,437 | 36,437 |
| **Coverage** | | | | | |
| Covered genome length (bp) | 962,980,503 | 1,188,042,476 | 1,343,670,392 | 1,444,818,443 | 1,516,374,355 |
| Genome coverage | 30.01% | 37.02% | 41.87% | 45.02% | 47.25% |
| **Gap** | | | | | |
| Inserted gap length (bp) | 1,277,354 | 2,060,052 | 2,532,043 | 2,622,840 | 2,758,438 |
|  | | | | | |
| **Sampling percentage** | **60%** | **70%** | **80%** | **90%** | **100%** |
| RNA-seq pair number | 68,293,014 | 79,675,183 | 91,057,352 | 102,439,521 | 113,821,682 |
| **Accuracy** | | | | | |
| Consistence | 6,070 | 6,312 | 6,529 | 6,750 | 6,942 |
| Inversions | 0 | 1 | 2 | 1 | 1 |
| Correctable relocations | 429 | 451 | 465 | 476 | 476 |
| Erroneous relocations | 228 | 240 | 249 | 252 | 256 |
| Translocations | 39 | 41 | 43 | 43 | 48 |
| Unknown | 3 | 3 | 4 | 4 | 4 |
| Total correct links^$^ | 6,499 | 6,763 | 6,994 | 7,226 | 7,418 |
| Total links | 6,769 | 7,048 | 7,292 | 7,526 | 7,727 |
| Accuracy | 96.01% | 95.96% | 95.91% | 96.01% | 96.00% |
| **Length** | | | | | |
| N50 length (bp) | 180,366 | 182,479 | 184,519 | 186,543 | 188,439 |
| Improvement | 21.28% | 22.70% | 24.08% | 25.44% | 26.71% |
| Corrected N50 length(bp) | 178,330 | 179,886 | 181,516 | 183,802 | 185,217 |
| Corrected improvement | 19.91% | 20.96% | 22.06% | 23.59% | 24.54% |
| **Sequence Number** | | | | | |
| P_RNA_scaffolder sequence number | 29,668 | 29,399 | 29,145 | 28,911 | 28,710 |
| Initial contigs number | 36,437 | 36,437 | 36,437 | 36,437 | 36,437 |
| **Coverage** | | | | | |
| Covered genome length (bp) | 1,581,752,119 | 1,635,117,388 | 1,680,858,159 | 1,719,821,831 | 1,752,174,258 |
| Genome coverage | 49.29% | 50.95% | 52.37% | 53.59% | 54.60% |
| **Gap** | | | | | |
| Inserted gap length (bp) | 2,894,586 | 3,013,075 | 3,169,841 | 3,404,701 | 3,606,780 |

^$^ The correct links are links of consistence and correctable relocations.

**Table S3. The performance of P_RNA_scaffolder with paired-end RNA-sequencing data from different tissues**

|  | **Brain** | **Liver** | **Lung** | **Brain&Liver** | **Brain&Liver&Lung** | **Brain&Liver&Lung&Cell** |
| --- | --- | --- | --- | --- | --- | --- |
| Clean RNA-seq pair number | 113,821,682 | 110,192,645 | 90,444,749 | 224,014,327 | 314,459,076 | 744,391,313 |
| **Accuracy** | | | | | | |
| Consistence | 6,942 | 5,764 | 5,735 | 8,161 | 8,828 | 10,251 |
| Inversions | 1 | 3 | 0 | 4 | 4 | 6 |
| Correctable relocations | 476 | 271 | 355 | 496 | 539 | 596 |
| Erroneous relocations | 256 | 90 | 120 | 251 | 260 | 278 |
| Translocations | 48 | 50 | 171 | 66 | 104 | 139 |
| Unknown | 4 | 2 | 5 | 5 | 9 | 12 |
| Total correct links^$^ | 7,418 | 6,035 | 6,090 | 8,657 | 9,367 | 10,847 |
| Total links | 7,727 | 6,180 | 6,386 | 8,983 | 9,744 | 11,282 |
| Accuracy | 96.00% | 97.65% | 95.36% | 96.37% | 96.13% | 96.14% |
| **Length** | | | | | | |
| N50 length (bp) | 188,439 | 174,870 | 176,202 | 204,493 | 225,101 | 279,888 |
| Improvement | 26.71% | 17.59% | 18.48% | 37.51% | 51.36% | 88.20% |
| Corrected N50 length(bp) | 185,217 | 173,820 | 174,472 | 198,714 | 211,422 | 262,548 |
| Corrected improvement | 24.54% | 16.88% | 17.32% | 33.62% | 42.17% | 76.54% |
| **Sequence Number** | | | | | | |
| P_RNA_scaffolder sequence number | 28,710 | 30,257 | 30,051 | 27,454 | 26,693 | 25,155 |
| Initial contigs number | 36,437 | 36,437 | 36,437 | 36,437 | 36,437 | 36,437 |
| **Coverage** | | | | | | |
| Covered genome region (bp) | 1,752,174,258 | 1,425,646,462 | 1,844,571,066 | 1,989,749,033 | 2,336,248,178 | 2,743,787,273 |
| Genome Coverage | 54.60% | 44.42% | 57.48% | 62.00% | 72.80% | 85.50% |

^$^ The correct links include links of consistence and correctable relocations.

**Table S4. The accuracy and performance of six scaffolders on human contigs**

|  | **P_RNA_scaffolder** | **L_RNA_scaffolder** | **AGOUTI*** | **BESST_RNA** | **Rascaf** | **RNAPATH*** |
| --- | --- | --- | --- | --- | --- | --- |
| Consistence | 6,942 | 3,658 | 4,268 | 6,259 | 4,185 | 4,352 |
| Inversions | 1 | 5 | 0 | 4 | 16 | 0 |
| Correctable relocations | 476 | 262 | 336 | 338 | 557 | 407 |
| Erroneous relocations | 256 | 90 | 212 | 194 | 257 | 293 |
| Translocations | 48 | 98 | 147 | 94 | 23 | 244 |
| Unknown | 4 | 1 | 16 | 48 | 2 | 19 |
| Total correct links^$^ | 7,418 | 3,920 | 4,604 | 6,597 | 4,742 | 4,759 |
| Total links | 7,727 | 4,114 | 4,979 | 6,913 | 5,040 | 5,315 |
| Accuracy | 96.00% | 95.28% | 92.47% | 95.43% | 94.09% | 89.54% |

^$^ The correct links include links of consistence and correctable relocations.

* AGOUTI and RNAPATH did not provide the orientation information of scaffolded contigs. Therefore, if two scaffolded contigs by AGOUTI or RNAPATH had the same order as hg38 assembly, we considered the connection to be consistent.

**Table S5. The runtime of six scaffolders on human contigs (minutes)**

| **P_RNA_scaffolder** | | **L_RNA_scaffolder** | | **AGOUTI** | | **BESST_RNA** | | **Rascaf** | | **RNAPATH** | |
| --- | --- | --- | --- | --- | --- | --- | --- | --- | --- | --- | --- |
| STEP | TIME | STEP | TIME | STEP | TIME | STEP | TIME | STEP | TIME | STEP | TIME |
| hisat-build | 103.71 | normalization | 205.28 | Augustus | 138.42 | hisat-build | 103.71 | hisat-build | 103.71 | Augustus | 138.42 |
| hisat2 | 32.77 | trinity | 2,462.32 | hisat-build | 103.71 | hisat2 | 32.77 | hisat2 | 32.77 | hisat-build | 103.71 |
| P_RNA_scaffolder | 58.55 | L_RNA_scaffolder | 89.83 | hisat2 | 32.77 | samtools view | 76.55 | samtools view | 76.55 | hisat2 | 32.77 |
| Total | 195.03 | Total | 2,757.43 | AGOUTI | 26.45 | samtools sort | 90.70 | samtools sort | 90.70 | joining-pairs generation* | 26.45 |
|  |  |  |  | Total | 301.35 | samtools index | 3.57 | Rascaf | 24.45 | RNAPATH | 44.13 |
|  |  |  |  |  |  | BESST_RNA | 17.13 | Rascaf-join | 6.06 | Total | 345.48 |
|  |  |  |  |  |  | Total | 324.43 | Total | 334.24 |  |  |

* The noise-free joining pairs were generated by AGOUTI following Zhang *et al.*’s strategy [[1](#_ENREF_1)].

**Table S6. The accuracy and performance of six scaffolders on E.coli contigs**

|  | **P_RNA_scaffolder** | **L_RNA_scaffolder** | **AGOUTI*** | **BESST_RNA** | **Rascaf** | **RNAPATH*** |
| --- | --- | --- | --- | --- | --- | --- |
| Consistence | 67 | 31 | 62 | 67 | 48 | 63 |
| Inversions | 0 | 0 | 2 | 0 | 3 | 0 |
| Correctable relocations | 0 | 0 | 0 | 0 | 0 | 0 |
| Erroneous relocations | 0 | 0 | 1 | 0 | 0 | 0 |
| Translocations | 0 | 0 | 0 | 0 | 0 | 0 |
| Total correct links^$^ | 67 | 31 | 64 | 67 | 48 | 63 |
| Total links | 67 | 31 | 65 | 67 | 51 | 63 |
| Accuracy | 100% | 100% | 98.46% | 100% | 94.12% | 100% |

^$^ The correct links include links of consistence and correctable relocations.

* AGOUTI and RNAPATH did not provide the orientation information of scaffolded contigs. Therefore, if two scaffolded contigs by AGOUTI or RNAPATH had the same order as E.coli reference assembly, we considered the connection to be consistent.

**Table S7. The runtime of six scaffolders on E.coli contigs (minutes)**

| **P_RNA_scaffolder** | | **L_RNA_scaffolder** | | **AGOUTI** | | **BESST_RNA** | | **Rascaf** | | **RNAPATH** | |
| --- | --- | --- | --- | --- | --- | --- | --- | --- | --- | --- | --- |
| STEP | TIME | STEP | TIME | STEP | TIME | STEP | TIME | STEP | TIME | STEP | TIME |
| bwa index | 0.07 | normalization | 59.14 | Augustus | 6.57 | bwa index | 0.07 | bwa index | 0.07 | Augustus | 6.57 |
| bwa mem | 9.87 | trinity | 160.5 | bwa index | 0.07 | bwa mem | 9.87 | bwa mem | 9.87 | bwa index | 0.07 |
| P_RNA_scaffolder | 9.13 | L_RNA_scaffolder | 0.13 | bwa mem | 9.87 | samtools view | 24.77 | samtools view | 24.77 | bwa mem | 9.87 |
| Total | 19.07 | Total | 219.77 | AGOUTI | 12.23 | samtools sort | 35.08 | samtools sort | 35.08 | joining-pairs generation* | 12.23 |
|  |  |  |  | Total | 28.74 | samtools index | 1.65 | Rascaf | 6.97 | RNAPATH | 0.03 |
|  |  |  |  |  |  | BESST_RNA | 6.38 | Rascaf-join | 0.001 | Total | 28.77 |
|  |  |  |  |  |  | Total | 77.82 | Total | 76.761 |  |  |

**Table S8. The accuracy and performance of six scaffolders on C.elegans contigs**

|  | **P_RNA_scaffolder** | **L_RNA_scaffolder** | **AGOUTI*** | **BESST_RNA** | **Rascaf** | **RNAPATH*** |
| --- | --- | --- | --- | --- | --- | --- |
| Consistence | 474 | 255 | 444 | 472 | 404 | 449 |
| Inversions | 0 | 1 | 6 | 0 | 0 | 4 |
| Correctable relocations | 5 | 3 | 5 | 5 | 3 | 5 |
| Erroneous relocations | 0 | 0 | 1 | 0 | 0 | 0 |
| Translocations | 1 | 0 | 2 | 1 | 0 | 3 |
| Total correct links^$^ | 479 | 258 | 449 | 477 | 407 | 454 |
| Total links | 480 | 259 | 458 | 478 | 407 | 461 |
| Accuracy | 99.7916% | 99.6138% | 98.0349% | 99.7907% | 100% | 98.4815% |

^$^ The correct links include links of consistence and correctable relocations.

* AGOUTI and RNAPATH did not provide the orientation information of scaffolded contigs. Therefore, if two scaffolded contigs by AGOUTI or RNAPATH had the same order as C.elegans reference assembly, we considered the connection to be consistent.

**Table S9. The runtime of six scaffolders on C.elegans contigs (minutes)**

| **P_RNA_scaffolder** | | **L_RNA_scaffolder** | | **AGOUTI** | | **BESST_RNA** | | **Rascaf** | | **RNAPATH** | |
| --- | --- | --- | --- | --- | --- | --- | --- | --- | --- | --- | --- |
| STEP | TIME | STEP | TIME | STEP | TIME | STEP | TIME | STEP | TIME | STEP | TIME |
| hisat-build | 2.03 | normalization | 27.33 | Augustus | 16.20 | hisat-build | 2.03 | hisat-build | 2.03 | Augustus | 16.20 |
| hisat2 | 5.56 | trinity | 172.26 | hisat-build | 2.03 | hisat2 | 5.56 | hisat2 | 5.56 | hisat-build | 2.03 |
| P_RNA_scaffolder | 5.18 | L_RNA_scaffolder | 4.18 | hisat2 | 5.56 | samtools view | 17.72 | samtools view | 17.72 | hisat2 | 5.56 |
| Total | 12.77 | Total | 203.77 | AGOUTI | 8.2 | samtools sort | 23.72 | samtools sort | 23.72 | joining-pairs generation* | 8.2 |
|  |  |  |  | Total | 31.99 | samtools index | 1.10 | Rascaf | 4.93 | RNAPATH | 0.20 |
|  |  |  |  |  |  | BESST_RNA | 3.93 | Rascaf-join | 0.18 | Total | 32.19 |
|  |  |  |  |  |  | Total | 54.06 | Total | 54.14 |  |  |

**Table S10. The improvement of genome assemblies from mate-pair scaffolding strategy by P_RNA_scaffoler**

| **Assemblies** | **N50 size before P_RNA_scaffolder**  **scaffolding (bp)** | **N50 size after P_RNA_scaffolder**  **Scaffolding (bp)** | **Improvement** |
| --- | --- | --- | --- |
| MIP scaffolder with 2K library | 142,981 | 282,935 | 197.88% |
| MIP scaffolder with 5K library | 144,426 | 289,369 | 200.36% |
| MIP scaffolder with 10K library | 716,072 | 1,590,900 | 222.17% |
| MIP scaffolder with 35K library | 329,194 | 680,762 | 206.80% |
| Opera with 2K library | 155,088 | 228,284 | 147.20% |
| Opera with 5K library | 261,091 | 366,358 | 140.32% |
| Opera with 10K library | 142,384 | 190,651 | 133.90% |
| Opera with 35K library | 3,726,998 | 4,031,677 | 108.17% |
| Soapdenovo with 2K library | 142,219 | 278,746 | 196.00% |
| Soapdenovo with 5K library | 162,188 | 364,609 | 224.81% |
| Soapdenovo with 10K library | 170,023 | 405,873 | 238.72% |
| Soapdenovo with 35K library | 754,023 | 1,318,453 | 174.86% |
| SOPRA with 2K library | 158,200 | 349,214 | 220.74% |
| SOPRA with 5K library | 266,210 | 646,557 | 242.87% |
| SOPRA with 10K library | 143,503 | 284,105 | 197.98% |
| SSPACE with 2K library | 142,480 | 280,463 | 196.84% |
| SSPACE with 5K library | 180,409 | 459,242 | 254.56% |
| SSPACE with10K library | 181,186 | 457,881 | 252.71% |
| SSPACE with 35K library | 1,457,909 | 2,722,476 | 186.74% |

**Table S11. The scaffolding performance of P_RNA_scaffolder with mouse paired-end RNA-sequencing reads on human contigs**

| Cleaned RNA-seq pair number | 38,268,760 |
| --- | --- |
| **Accuracy** | |
| Consistence | 47 |
| Inversions | 0 |
| Correctable relocations | 6 |
| Erroneous relocations | 3 |
| Translocations | 29 |
| Total correct links^$^ | 53 |
| Total links | 85 |
| Accuracy | 62.35% |
| **Improvement** | |
| N50 length (bp) | 149,028 |
| Improvement | 0.21% |
| Corrected N50 length(bp) | 148,930 |
| Corrected improvement | 0.14% |
| **Sequence number** | |
| P_RNA_scaffolder sequence number | 36,352 |
| Initial contigs number | 36,437 |
| **Coverage** | |
| Coverage length (bp) | 147,137,931 |
| Coverage | 4.58% |
| Gap length (bp) | 258,813 |

^$^ The correct links include links of consistence and correctable relocations.

**Table S12. The performance of P_RNA_scaffolder with BLAT alignment and without BLAT alignment**

|  | **with BLAT alignment** | **without BLAT alignment** |
| --- | --- | --- |
| Consistence | 6,942 | 7,358 |
| Inversions | 1 | 12 |
| Correctable relocations | 476 | 473 |
| Erroneous relocations | 256 | 333 |
| Translocations | 48 | 141 |
| Unknown | 4 | 53 |
| Total correct links^$^ | 7,418 | 7,831 |
| Total links | 7,727 | 8,370 |
| Accuracy | 96.00% | 93.56% |

**References**

1. Zhang SV, Zhuo L, Hahn MW: **AGOUTI: improving genome assembly and annotation using transcriptome data**. *Gigascience* 2016, **5**(1):31.
