# Supplementary material for: P_RNA_scaffolder: a fast and accurate genome scaffolder using paired-end RNA-sequencing reads
Source: BMC Genomics. 2018 Mar 2;19:175. doi: 10.1186/s12864-018-4567-3 (PMC5834899; doi:10.1186/s12864-018-4567-3)
Supplement: Supplementary file 2 — Figure S1. Flow chart of P_RNA_scaffolder. Figure S2. The correlation between corrected N50 size improvement and genome coverage. Figure S3. Accuracy and N50 sizes of P_RNA_scaffolder and other mate-pair scaffolder. Figure S4. Influence of alternative splicing on the accuracy of P_RNA_scaffolder. (DOCX 399 kb) [file 12864_2018_4567_MOESM2_ESM.docx]

**Supplementary Figures**

**Figure S1. Flow chart of P_RNA_scaffolder**


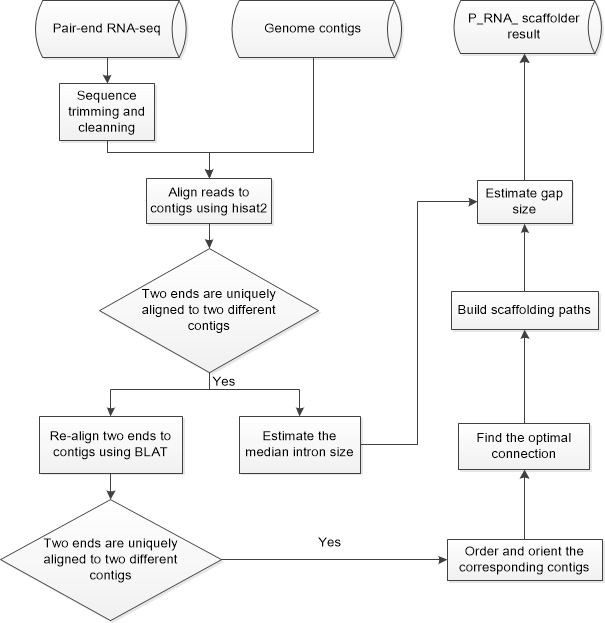


**Figure S2. The correlation between corrected N50 size improvement and genome coverage**

(a) The correlation between corrected N50 size improvements and genome coverages during sampling brain RNA-sequencing reads. (b) The correlation between corrected N50 size improvement and genome coverage in different tissues.


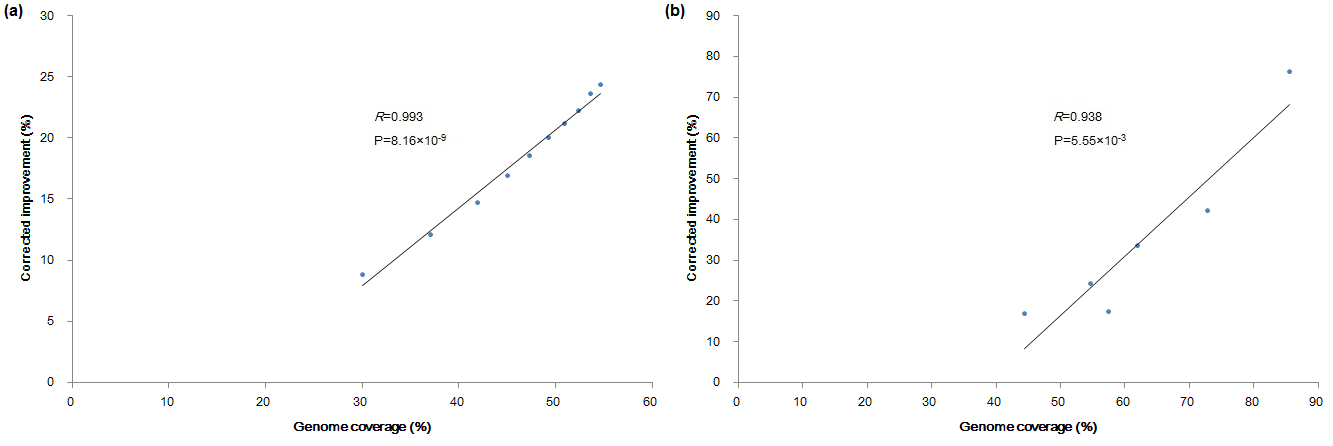


**Figure S3. Accuracy and N50 sizes of P_RNA_scaffolder and other mate-pair scaffolder**


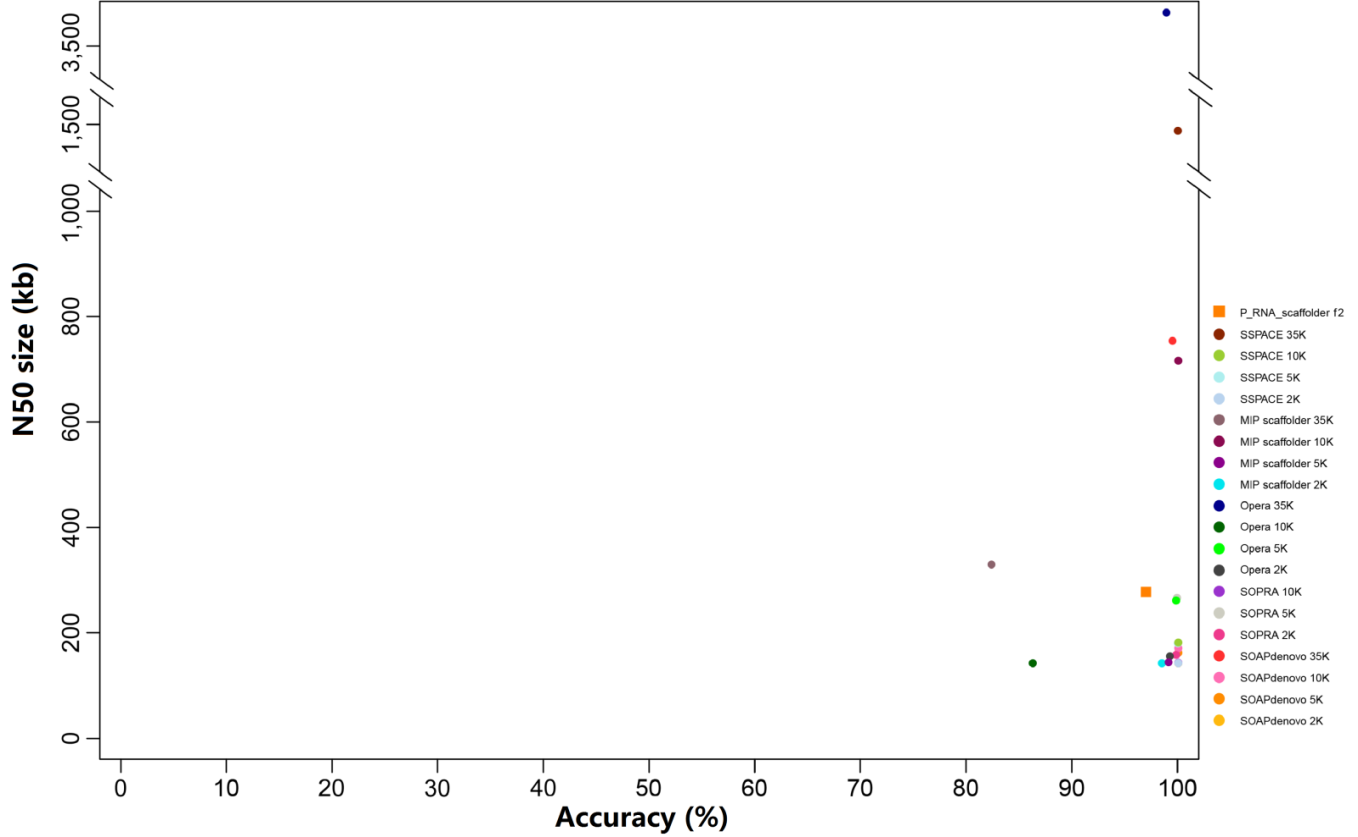


**Figure S4. Influence of alternative splicing on the accuracy of P_RNA_scaffolder**

(a)


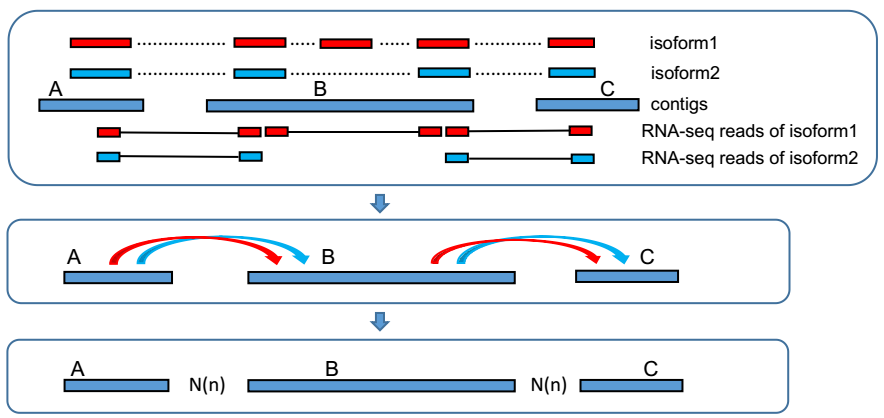


(b)


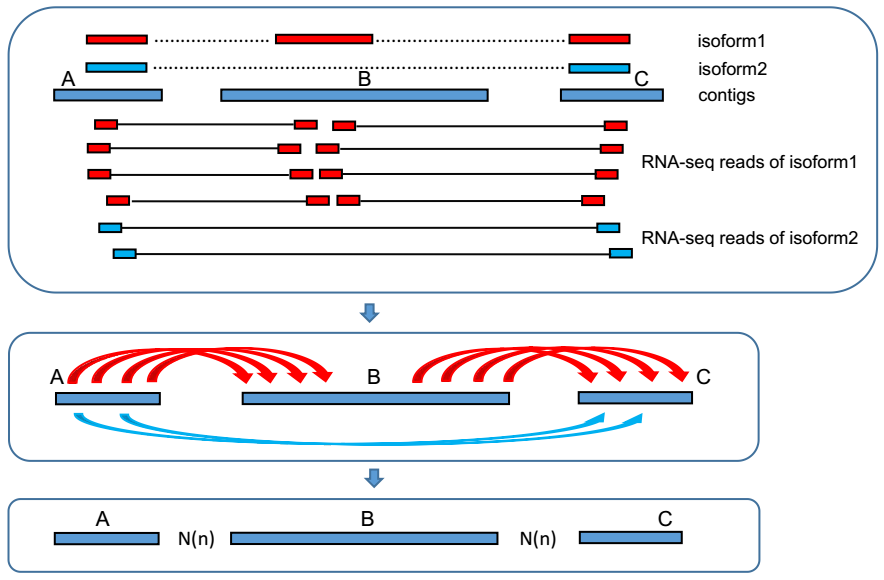


(c)


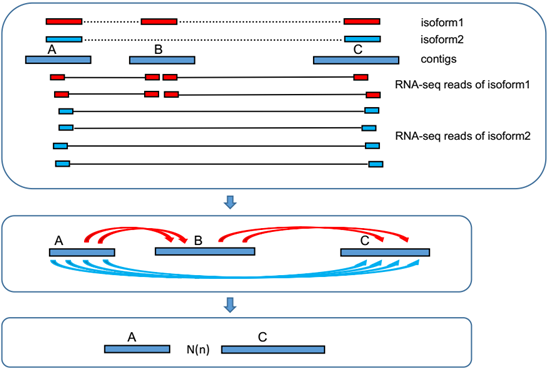


The A, B, and C are the genome sequences. The alternative splicing isoforms are colored and displayed above the genome sequences, where the boxes are exons and the dash lines are introns. The paired-end RNA-sequencing reads are displayed below the genome sequences. The reads have the same color to the corresponding isoforms. (**a**) All isoforms are aligned to the contigs (A, B and C). The built scaffold consists of these contigs and could completely cover all isoforms. (**b**) The dominantly expressed isoform (isoform 1) includes all exons of this gene. The built scaffold based on the reads of this isoform also completely covers all isoforms. (**c**) The dominantly expressed isoform (isoform 2) include a part of exons. The alternative exonic contig (C) is not scaffolded.
